# Formulation of a Novel Hesperetin-Loaded Nanoemulsion and Its Promising Effect on Osteogenesis

**DOI:** 10.3390/pharmaceutics16060698

**Published:** 2024-05-23

**Authors:** Maria Júlia Mancim-Imbriani, Jonatas Lobato Duarte, Leonardo Delello Di Filippo, Letícia Pereira Lima Durão, Marlus Chorilli, Denise Madalena Palomari Spolidorio, Patricia Milagros Maquera-Huacho

**Affiliations:** 1 Department of Diagnosis and Surgery, São Paulo State University (UNESP), School of Dentistry, Araraquara CEP 14801-385, São Paulo, Brazil; maria.mancim@unesp.br (M.J.M.-I.); leticia.durao@unesp.br (L.P.L.D.); 2 Department of Physiology and Pathology, São Paulo State University (UNESP), School of Dentistry, Araraquara CEP 14801-385, São Paulo, Brazil; denise.mp.spolidorio@unesp.br; 3 Department of Drugs and Medicines, São Paulo State University (UNESP), School of Pharmaceutical Sciences, Araraquara CEP 14800-903, São Paulo, Brazil; jl.duarte@unesp.br (J.L.D.); leonardo.filippo@unesp.br (L.D.D.F.); marlus.chorilli@unesp.br (M.C.)

**Keywords:** nanoparticles, flavonoids, materials testing, cytotoxicity tests, bone regeneration, hesperetin

## Abstract

Alternative therapies associating natural products and nanobiotechnology show new perspectives on controlled drug release. In this context, nanoemulsions (NEs) present promising results for their structural design and properties. Hesperetin (HT), a flavonoid mainly found in citrus fruits, presents highlighted bone benefits. In this context, we developed a hesperetin-loaded nanoemulsion (HT-NE) by sonication method and characterized it by dynamic light scattering, analyzing its encapsulation efficiency, and cumulative release. The biocompatibility in human osteoblasts Saos-2-like was evaluated by the cytotoxicity assay and IC_50_. Then, the effects of the HT-NE on osteogenesis were evaluated by the cellular proliferation, calcium nodule formation, bone regulators gene expression, collagen quantification, and alkaline phosphatase activity. The results showed that the formulation presented ideal values of droplet size, polydispersity index, and zeta potential, and the encapsulation efficiency was 74.07 ± 5.33%, showing a gradual and controlled release. Finally, HT-NE was shown to be biocompatible and increased cellular proliferation, and calcium nodule formation, regulated the expression of *Runx2*, *ALPL*, and *TGF-β* genes, and increased the collagen formation and alkaline phosphatase activity. Therefore, the formulation of this NE encapsulated the HT appropriately, allowing the increasing of its effects on mechanisms to improve or accelerate the osteogenesis process.

## 1. Introduction

Nowadays, we have a wide array of conditions presenting bone loss, including periodontal diseases, osteoporosis, and fractures in the bone [1], so the search for treatments for these diseases is growing. Several treatments, medicines, and biomaterials are proposed but, in some cases, are not enough to restore bone formation [2]. On the other hand, several disadvantages and side effects have been observed, for example, bisphosphonate is used for the treatment of osteoporosis, but its use can cause bone necrosis [3].

In recent years, there has been an increasing focus on drug discovery and development incorporating drug delivery systems. Among these, nanoparticles offer several advantages due to their higher surface-to-mass ratio and diverse quantum properties [4,5]. In addition to facilitating drug release, nanoparticles can exhibit other favorable characteristics such as enhancing solubility and enabling more selective distribution, thereby reducing adverse effects [6,7,8,9,10]. Nanoemulsions (NE) are a type of nanoparticle comprising an oil phase (non-polar) and an aqueous phase (polar) stabilized by an emulsifier. They show promising potential as drug delivery systems, controlling release and directing it to the target site [11]. Furthermore, NE can effectively carry lipophilic pharmaceutical compounds, shielding them from external factors such as pH, oxidation, and hydrolysis, and they have demonstrated greater resistance to flocculation and coalescence compared to conventional emulsions [12].

Flavonoids are bioactive compounds that belong to the polyphenol group, they can be classified as flavonols, flavones, flavonones, flavon-3-ol, isoflavonones, and anthocyanins [13]. Hesperetin (HT) is a metabolite of hesperidin, belonging to the flavonone subgroup [14]. It can be found mainly in citric fruits, like oranges and grapefruit [14]. Several studies suggested the biological properties of HT like analgesic, anti-cancer, anti-inflammatory, antioxidant, and neuroprotector effects [15,16,17]. Among these properties, beneficial effects on bone metabolism are highlighted [15,16,17,18].

HT presents characteristics that can improve bone mineral density through different mechanisms [19]. Trzeciakiewicz et al. [20], 2010, suggested that this flavonoid can stimulate the differentiation of primary osteoblasts in rats, by Smad-dependent BMP (bone morphogenetic protein) and MAPK signaling pathways. HT can enhance the expression of bone metabolism genes, like *ALPL*, *Runx2*, *OCN*, *Osterix*, *osteopontin* (*OPN*), and *COL1A1* in osteoblasts and mesenchymal cells [20,21]. Also, in high glucose conditions, HT can promote osteoblastic differentiation [22]. In this context, HT suppresses the osteogenesis inhibitor factors through the activation of the PI3K/Akt pathway and suppression of ROS [23]. Additionally, Liu et al. [24] suggest that HT is able to suppress glucocorticoid effects through the regulation of the ERK signaling pathway. However, HT is a hydrophobic compound, for this reason, it is necessary to dilute it in other solubilizing agents, sometimes toxic [25].

Given that HT is a flavonoid with benefic and promising properties specifically on bone metabolism, this study aimed to formulate a new hesperetin-loaded nanoemulsion (HT-NE) and evaluate its colloidal stability and the in vitro effect on osteogenesis. To the best of our knowledge, this is the first manuscript investigating the effects of HT encapsulated in an NE for biomedical applications as bone tissue regeneration in osteoblasts.

## 2. Materials and Methods

### 2.1. Nanoemulsion Development and Characterization

#### 2.1.1. Nanoemulsion Preparation

The formulations were prepared based on Bouchemal et al. [26] and Bonifácio et al. [27] methodologies with modifications. The oil phase was prepared with soy phosphatidylcholine (Lipoid, Ludwigshafen, Germany) (0.4% *w*/*v*), capric/caprylic acid triglycerides (KLK, São Paulo, SP, Brazil) (1.8% *w*/*v*), polysorbate 80 (Deg, São Paulo, Brazil) (2.1% *w*/*v*), and 100 mM of HT (Sigma-Aldrich, Barueri, SP, Brazil). The aqueous phase was composed of ultra-pure water that was added dropwise to the oil phase, resulting in a pre-emulsion. In sequence, this pre-emulsion was sonicated for 2 min in a sonicator (Qsonica, Newtown, CT, USA), with a pulse on of 1 min and pulse off for 30 s, and an amplitude of 35. The blank formulation was prepared as described, without the HT addition (Blank NE).

#### 2.1.2. Droplet Size, Polydispersity Index, and Zeta Potential

The droplet size and polydispersity index (PDI) were obtained using the dynamic light scattering (DLS) technique and the zeta potential was measured by electrophoretic mobility, which was performed on Zetasizer equipment (Nano ZS, Malvern, UK). The formulations were diluted in deionized water (1:25), and the analysis of stability was performed in triplicate for 1, 7, 21, and 90 days to evaluate the HT-NE. Additionally, an evaluation of 1 day was performed on the Blank NE to check the similarity between the formulations.

#### 2.1.3. Analytic Methodology for Hesperetin Quantification

The high-performance liquid chromatography (HPLC) analysis was performed in an HPLC Agilent 1220 infinity system (Agilent, Santa Clara, CA, USA) equipped with a column Synergy (150 × 4.6 mm, 4 µm). The volume of injection was 20 μL with a 1.0 mL/min flow. The mobile phase was constituted in acetonitrile/acidified water with acetic acid 0.1% (50:50, *v*/*v*). The detector was fixed at 290 nm for chromatogram acquisition.

For the analytical curve, a standard solution (2000 μg/mL) of HT in acetonitrile (J.T. Baker, Phillipsburg, NJ, USA) was prepared. The analytic curve was performed in triplicate at the following concentrations: 25, 75, 125, 250, 375, 500, and 750 µg/mL of HT in pure acetonitrile. Three independent analytic curves were created, and the linearity was obtained by linear regression using the minimum squares method.

#### 2.1.4. Encapsulation Efficiency

The encapsulation efficiency of HT on the NE was calculated by the difference between the mass of encapsulated HT and total HT mass. Briefly, 1 mL of the formulation was submitted to centrifugation of 14,000 rpm for 30 min to precipitate the Free HT. A supernatant aliquot of 100 µL was added to a flask containing 900 µL of acetonitrile and sonicated (amplitude 35, during 1 min) to disrupt the NE. The HT quantification was performed by HPLC as described previously.

#### 2.1.5. In Vitro Release Assay

The in vitro release assay was conducted under sink conditions. Experiments were performed using modified Franz cells, featuring a diffusion area of 1.77 cm^2^, within a Microette equipment (Hanson Research, Austin, TX, USA) and a polyethersulfone membrane (Sigma-Aldrich, Barueri, SP, Brazil). The receptor compartment of modified Franz cells was filled in with 7.0 mL of PBS (phosphate buffer saline) pH = 7.4.

The receptor solution was kept in a continuous shake at 300 rpm and room temperature (37 ± 2 °C) through a warm bath. For the evaluation of Free HT release, it was dissolved in a PBS with 1% polysorbate 80 solution. The quantification of HT released from the NE and in the free form was evaluated after 30 min, 1, 2, 4, 6, 8, and 10 h. The experiments were performed in triplicates and the quantification was performed by high-performance liquid chromatography (HPLC).

### 2.2. In Vitro Analyses in Osteoblasts Saos-2-like

#### 2.2.1. Cell Culture

Human osteoblasts cell line Saos-2-like, purchased from American Type Culture Collection (ATCC hTB-85), were cultured in Dulbecco’s Modified Eagle Medium (DMEM) supplemented with 10% fetal bovine serum (FBS), 100 IU mL^−1^ penicillin, and 100 μg/mL streptomycin (Gibco, Waltham, MA, USA). The cell culture was incubated at 37 °C in a humidified incubator containing 5% CO_2_.

The osteogenic medium was the normal medium supplemented with 50 mg/mL of ascorbic acid (Sigma-Aldrich, Barueri, SP, Brazil) and 10 mM of β-glycerolphosphate (Sigma-Aldrich, Barueri, SP, Brazil) [28].

#### 2.2.2. Cytotoxicity Assay

Previously, HT ≥ 98% (Sigma-Aldrich, Barueri, SP, Brazil) was dissolved in dimethylsulfoxide (DMSO) at a concentration of 1 M and stored at −20 °C to the obtention of desired concentrations. The final concentration of DMSO in the assay was less than 0.1% [29].

Human osteoblasts cell line Saos-2-like were seeded in a 96-well plate (5 × 10^4^ cell/well) and incubated for 24 h. Thereafter, the culture medium of the confluent cell monolayer was replaced by Free HT, HT-NE, Blank NE concentrations (8200 µM–0.5 µM *v*/*v*), Camptothecin 10 μM (positive control group) and culture medium (negative control group). The plate was incubated for 7 days at 37 °C in a humidified atmosphere containing 5% of CO_2_ and the spent medium was refreshed every 48 h. After 7 days, the cytotoxic effect was evaluated by methyl tetrazolium (MTT) (Sigma-Aldrich, St. Louis, MO, USA) assay.

The colorimetric analysis with MTT evaluates the cell metabolism through the cytochemical activity of the succinic dehydrogenase (SDH) enzyme, which represents the mitochondrial breath rate of viable cells [30]. Each well of the experimental and control groups received a solution containing 90 µL of culture medium and 10 µL of MTT solution, which was prepared with 5 mg of MTT powder and 1 mL of sterile PBS. The cells were incubated for 4 h at 37 °C and 5% CO_2_. Then, the culture medium with MTT was aspirated, and 100 µL of acidified isopropanol (HCl 0.04 N) was applied in each well. The cell viability was evaluated considering that cells with normal mitochondrial activity were colored with an intense violet. Optical density was read at 570 nm wavelength in a spectrophotometer (Synergy H1 Multi-Mode Reader-BioTek, Wilmington, DE, USA).

Finally, IC_50_ (half-maximum inhibitory concentration) is the concentration of the treatments at an inhibition ratio of 50%. Considering this, the IC_50_ values were measured using the software GraphPad Prism 9, where the X values are the logarithm (log) of concentration and Y values are the log of C/C0 × 100%, where C is each concentration’s optical densities and C0 is the control group’s optical density obtained by MTT assay.

#### 2.2.3. Cell Proliferation Analysis

To analyze cell proliferation, osteoblasts Saos-2-like were seeded in 48 wells plates at 1 × 10^4^ cells/well. After 24 h, the medium was refreshed with non-cytotoxic concentrations of HT-NE. The plate was incubated at 37 °C and 5% CO_2_ and the proliferative effect was evaluated after 1, 3, 5 and 7 days by Alamar Blue assay^®^, which is based on the capacity of metabolic active cells to convert the reagent in a fluorescent and colorimetric indicator. So, at the end of each period evaluated, the wells received the Alamar Blue^®^ solution 10% diluted in culture medium and the plates were incubated for 4 h in dark conditions. After this time, an aliquot of 100 µL of each well was transferred to a new 96-well plate, to be read in a spectrophotometer (Synergy H1 Multi-Mode Reader-BioTek, Wilmington, DE, USA) with wavelengths of 570 nm and 600 nm.

#### 2.2.4. Mineralized Nodules Formation

To evaluate the mineralized nodules deposited by the cells it was performed the alizarin red staining after 7 days of treatment. This way, the cells were seeded in 96-well plates, at 1 × 10^4^ cell/well. After 24 h the culture medium was refreshed for a new one containing non-cytotoxic concentrations of HT-NE, previously prepared in osteogenic medium. While the control group just received culture medium. The medium was refreshed every 48 h. After 7 days, the attached cells were washed two times with PBS with a pH of 7.2 and fixed with 100 μL ethanol 70% for 30 min. Then, each well was washed with distilled water two times and stained with 150 μL of alizarin red stain solution (40 mM, pH 4.2—Sigma-Aldrich, Barueri, SP, Brazil). The plates were kept in contact with the stain solution for 20 min at room temperature and slow agitation (300 rpm) (VDRL Shaker, Biomixer, São Paulo, SP, Brazil). Then, the wells were washed with distilled water until the excess stain solution was removed. The mineralized nodules were dissolved with 200 μL of cetylpyridine 10% (Sigma-Aldrich, Barueri, SP, Brazil) for 15 min under slow agitation (300 rpm) and room temperature conditions. Then, the absorbance was measured in a microplate reader (Synergy H1 Multi-Mode Reader-BioTek, Wilmington, DE, USA) at 562 nm.

#### 2.2.5. Bone Metabolism Gene Expression Quantification

Cells were seeded in a 12-well plate, at 9 × 10^4^ cells/well and treated with non-cytotoxic concentrations of HT-NE. After 1, 3, and 7 days, the cellular lysis and purification were performed using the RNeasy mini kit (Qiagen GmbH, Hilden, Germany) following the manufacturer protocol. The RNAm were quantified using the Synergy H1 equipment (Biotek, Wilmington, DE, USA) and RNAm of each sample (800 ng/mL) was transcript in cDNA using a thermocycler and the High-Capacity cDNA Reverse Transcriptions Kit (Applied Biosystems, Waltham, MA, USA), following the manufacturer’s protocol.

The cDNAs obtained were used in real-time PCR reactions to determine the relative amount of *Runx2* (Hs00231692_m1), *ALPL* (Hs01029144_m1), and *TGF-β* (Hs00248373_m1) genes. The relative abundance was measured by reverse transcription in real-time PCR (RT-qPCR) using Taqman, primer, and specific probe (TaqMan Gene Expression Assays, Applied Biosystems, Waltham, MA, USA) in a thermocycler StepOne Plus Real-Time PCR System (Applied Biosystems, Waltham, MA, USA). The reactions were performed in 96-well plates with a 20 μL final volume, including Taqman Universal PCR Master Mix (Applied Biosystems, Waltham, MA USA), Taqman Gene Expression Assay (Applied Biosystems, Waltham, MA, USA) to gene and model cDNA. The optimized thermocycler conditions were: 50 °C for 2 min, 95 °C for 10 min, followed by 40 cycles at 95 °C for 15 s and 60 °C for 1 min. The normalization was accomplished by the *GAPDH* (Hs02786624_g1) expression, which was not modified by experimental conditions. To compare the expression levels between the samples, the relative gene expression level was calculated using ΔΔCT comparative method with the thermocycler software StepOne Plus v2.1.

#### 2.2.6. Collagen Production by Picrosirius Red

After 3 and 7 days of treatment with non-cytotoxic concentrations of HT-NE, the supernatants were collected to evaluate the collagen quantification. Picrosirius Red was added to the samples in a 1:1 proportion, then they were kept in an Eppendorf ThermoMixer equipment at 400 rpm and 25 °C for 1 h. After this period the samples were centrifuged at 12,000 rpm and 25 °C for 10 min. The supernatants were thrown away and HCl 0.01 M was added to each sample, a new centrifugation was performed at 12,000 rpm and 25 °C for 10 min. Finally, the supernatants were thrown away, and the samples dissolved in NaOH 0.5 M. The absorbance was read in a spectrophotometer (Synergy H1 Multi-Mode Reader-BioTek, Wilmington, DE, USA) at 555 nm wavelength.

#### 2.2.7. Alkaline Phosphatase Activity

After the periods of osteogenic induction, at 3 days and 7 days, the alkaline phosphatase (ALP) activity was measured using the alkaline phosphatase kit (ABCAM, Boston, MA, USA) following the manufacturer’s instructions. Briefly, the samples and the substrate of pNPP (p-nitrophenyl phosphate) were mixed and reacted for 60 min, then the reaction was interrupted with the Stop solution. The absorbance was read in a spectrophotometer (Synergy H1 Multi-Mode Reader-BioTek, Wilmington, DE, USA) with a wavelength of 405 nm and the ALP activity was calculated based on the standard curve graph.

### 2.3. Statistical Analyses

All assays in this study were performed in triplicates and three independent experiments. The numerical data, obtained by applying the laboratory protocols, were subjected to specific statistical analysis using Turkey’s test and normalized through the Shapiro–Wilk normality test by the GraphPad Prism 9 software, and to all tests in this study a significance level of 5% (*p* < 0.05) was applied.

## 3. Results

### 3.1. Nanoemulsion Development and Characterization

#### 3.1.1. Droplet Size, Polydispersity Index, and Zeta Potential

**Figure 1:**
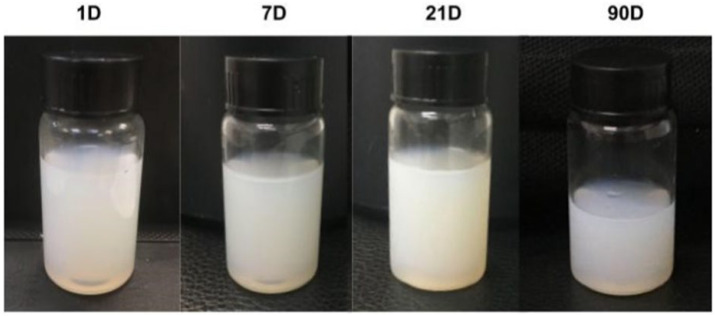
Representative images of HT-NE in different analyzed periods (1, 7, 21, and 90 days).

The HT-NE showed a translucence or slight turbidity of its surface during all periods under analysis (Figure 1), a common characteristic of this type of nanoparticle due to the small size of its droplets.

Table 1 shows the results of HT-NE characterization by DLS. It is possible to note that the droplet size was kept stable during the evaluated periods, 1 to 90 days, presenting an average value of around 89.41 ± 5.81 nm, when observed in all periods. The PDI presented a stable value on average of 0.267 ± 0.008 for the periods until 21 days, and a value of 0.584 ± 0.035 was obtained after 90 days. HT-NE zeta potential presented values higher than −20 mV and less than 20 mV for all the periods. The results of Blank NE were analyzed for 1 day and are also shown in this Table. It was demonstrated that the Blank NE presents a droplet size value of 97.86 ± 0.50 nm and a polydispersity index of 0.278 ± 0.003, similar to HT-NE. On the other hand, the Blank NE showed a zeta potential value of −26.9 ± 2.4 mV.

#### 3.1.2. Encapsulation Efficiency

The results of the encapsulation efficiency were given in percentage. The average obtained from three independent assays executed in triplicates was 74.07% with a standard deviation of 5.33%.

#### 3.1.3. In Vitro Release Assay

**Figure 2:**
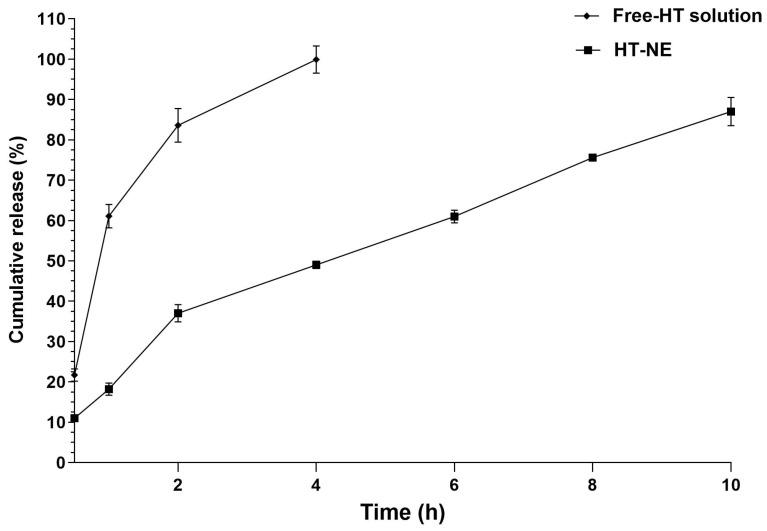
In vitro cumulative release of HT in a solution and loaded in the NE.

Figure 2 represents the release assay results. Suggesting a controlled and graduated release profile for both formulations, Free HT solution and HT-NE (Figure 2). The Free HT solution showed a fast release, with 21% of HT released after 30 min, reaching around 100% release in 4 h of experiment. On the other way, the NE showed a slower release rate, releasing 11% of HT after 30 min, 49% after 4 h, and 87% after 10 h of the experiment.

Table 2 presents the mathematical release kinetics models for HT-NE. The obtained results from HT through the NE show a proper adjustment to the Korsmeyer–Peppas model, a frequently used equation to describe the release kinetics to pharmaceuticals through controlled release systems. In this case. the k value was calculated as 18.21, and the obtained n value was 0.7485.

### 3.2. In Vitro Analyses in Osteoblasts Saos-2-like

#### 3.2.1. Cytotoxicity

**Figure 3:**
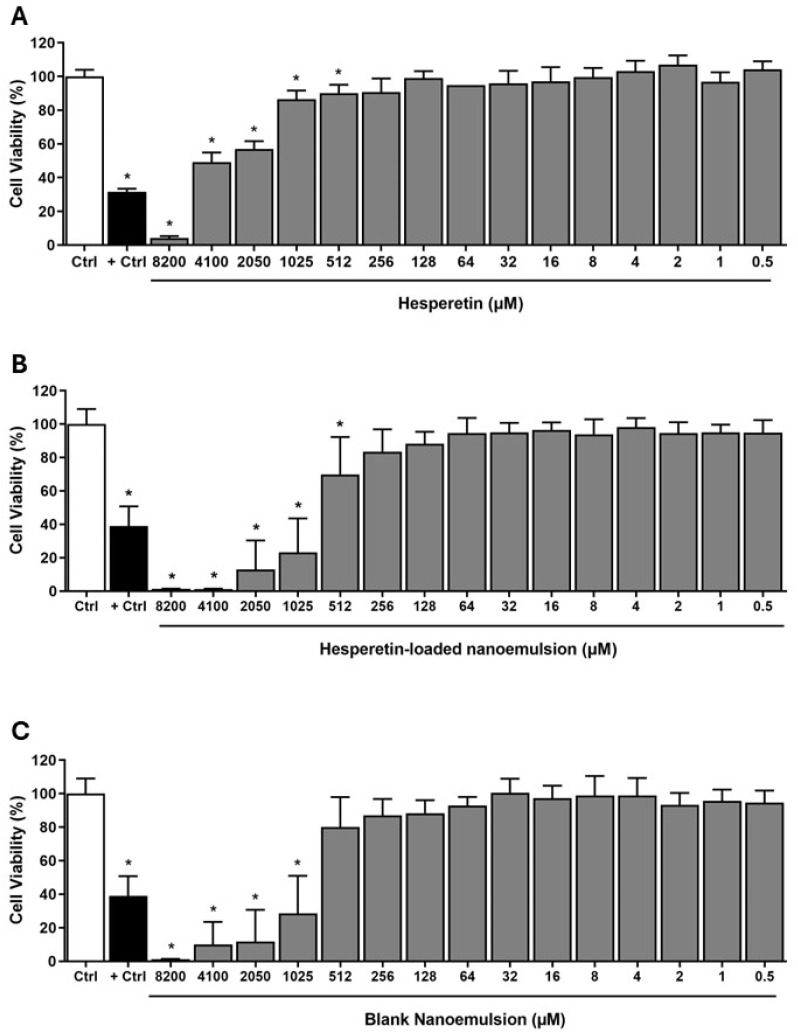
Cell viability percentage of osteoblasts Saos-2 after 7 days of treatment with different concentrations of Free HT (**A**), HT-NE (**B**), and Blank NE (**C**). Asterisk: Difference statistically significant compared with the negative control group (ANOVA, * *p* < 0.001).

Figure 3 shows the results of the cytotoxicity assay. The treatment with Free HT and HT-NE in concentrations greater than 512 µM showed a statistically significant decrease in osteoblasts cells after exposure for 7 days in comparison to the negative control group (*p* < 0.001). On the other hand, Blank NE showed a cytotoxic effect in concentrations greater than 1025 µM in comparison to the negative control group (*p* < 0.001).

#### 3.2.2. IC_50_ Value

**Figure 4:**
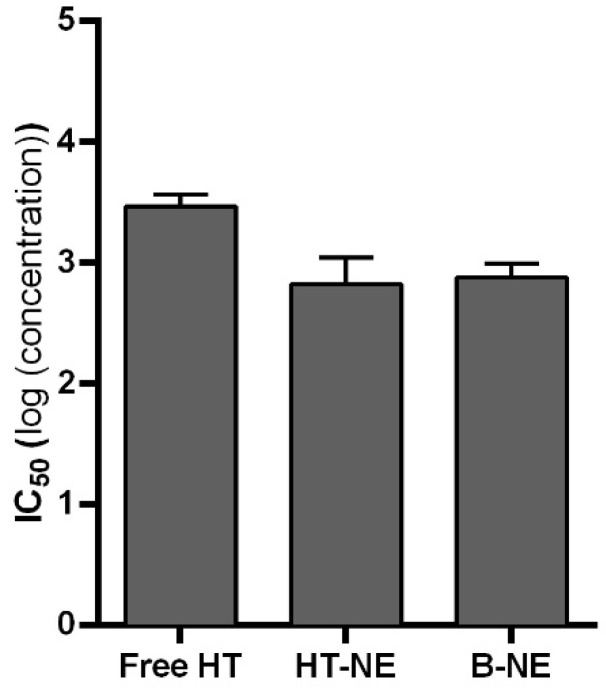
The IC_50_ value of the Saos-2-like cell line treated with Free HT, HT-NE, and Blank NE (ANOVA, *p* < 0.05).

Figure 4 shows the IC50 values of the treatments: Free HT and NE (HT-NE and Blank NE). The IC50 values were 3.46 log, 2.82 log, and 2.87 log (concentrations) to Free HT, HT-NE, and Blank NE, respectively.

Taking this into account, it is possible to note that Free HT presented an IC50 value higher than the NE tested.

#### 3.2.3. Cell Proliferation Analysis

**Figure 5:**
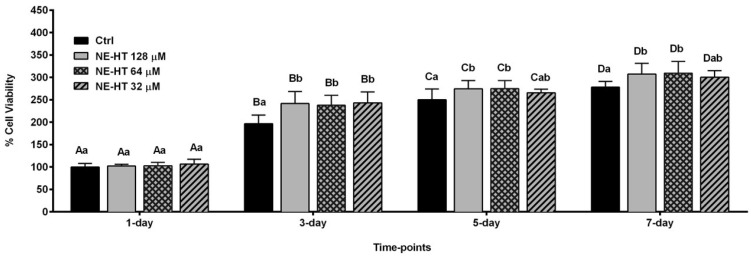
HT-NE effect on osteoblasts Saos-2 proliferation after treatment with non-cytotoxic concentrations (32 µM, 64 µM, and 128 µM) for 1, 3, 5, and 7 days. The same letters represent no statistical difference, different letters indicate statistical difference. The upper case indicates a comparison between periods and the lower case indicates a comparison between groups in the same period. (Two-way ANOVA, *p <* 0.05).

Figure 5 shows the cell proliferation results. It is possible to observe an increase in all groups when they are compared between different periods, showing a statistically significant difference (*p* < 0.05). On the first day of evaluation the groups did not present a statistical difference between them (*p* > 0.05), however, in 3, 5, and 7 days of evaluation the groups treated with HT-NE showed a statistically significant increase in cell proliferation when compared to the control group (*p* < 0.05). An interesting point was that at 5 and 7 days, the HT-NE treated groups with 128 µM and 64 µM concentrations, showed an increase in cell proliferation and showed a statistical difference compared to the control and HT-NE 32 µM groups (*p* < 0.05).

#### 3.2.4. Mineralized Nodules Formation

**Figure 6:**
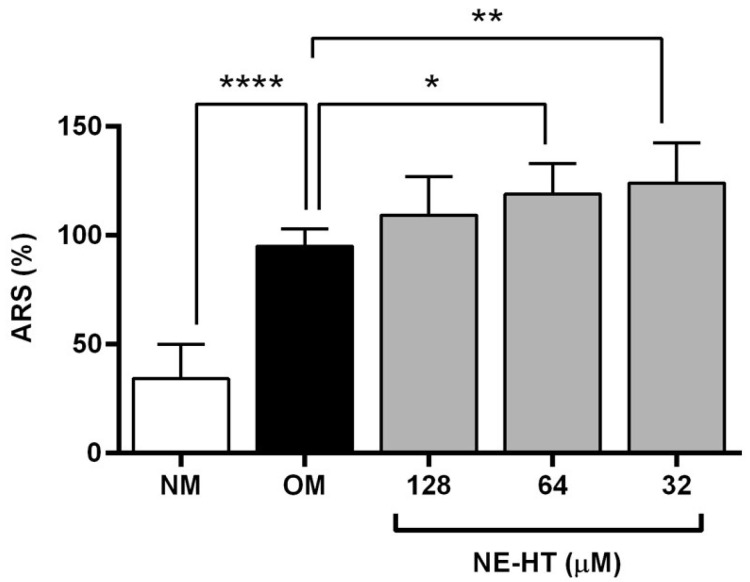
Calcium nodule formation quantification in human osteoblasts Saos-2 like after treatment with non-cytotoxic HT-NE (128 µM, 64 µM, and 32 µM) concentrations for 7 days. Measurement in the percentage of alizarin red absorbance. (ANOVA, α = 0.05). ┌─┐ represents statistically significant difference compared to osteogenic group (* *p* < 0.05, ** *p* < 0.01, **** *p* < 0.0001). NM = normal medium, OM = osteogenic medium.

Figure 6 describes the results of mineralized nodule formation. HT-NE treated groups at 32 µM and 64 µM showed a statistically significant increase in mineralized nodule formation when compared with the group treated with osteogenic medium only (*p* < 0.05, *p* < 0.01). However, the HT-NE 128 µM did not present a statistically significant difference (*p* > 0.05). However, we can observe that all groups treated with HT-NE showed an increase in mineralized nodule formation compared to the group that just received a normal medium (*p* < 0.0001).

#### 3.2.5. Bone Metabolism Gene Expression Quantification

**Figure 7:**
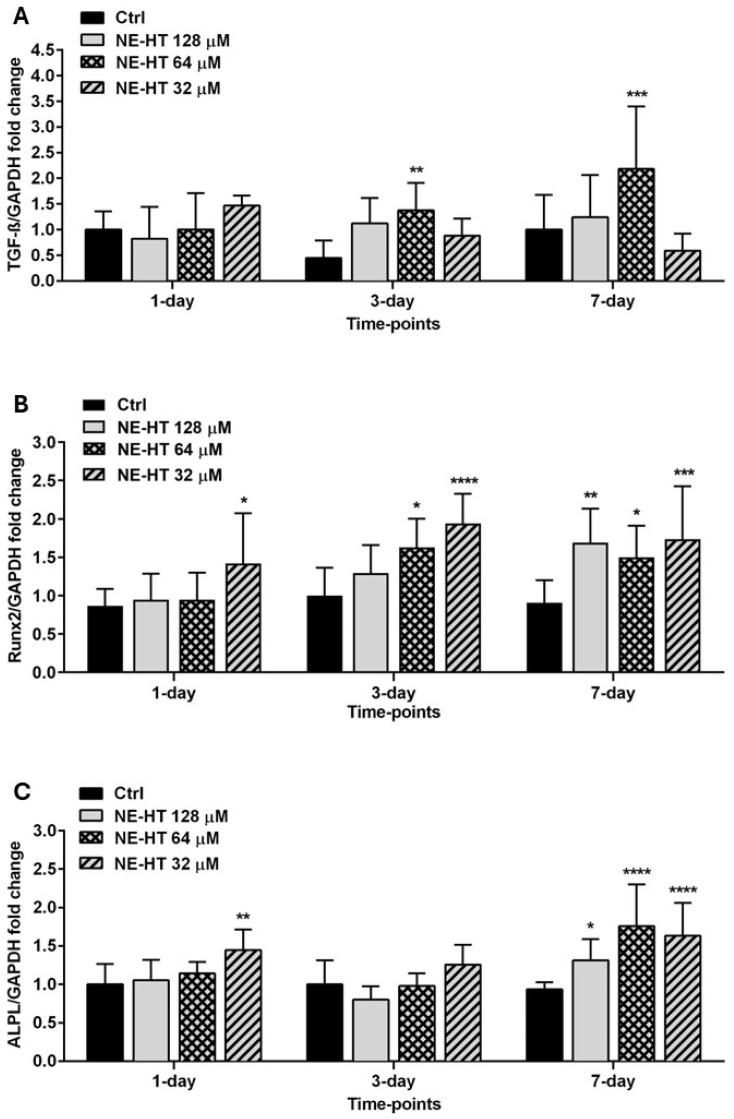
Gene expression levels of TGF-β (**A**), Runx2 (**B**) e ALPL (**C**) in human osteoblasts Saos-2 treated with non-cytotoxic HT-NE (128 µM, 64 µM and 32 µM) after 1, 3 and 7 days. Asterisk: Represents a statistically significant difference compared to the control group (one-way ANOVA, * *p* < 0.05, ** *p* < 0.01, *** *p* < 0.001, **** *p* < 0.0001).

Figure 7 represents the gene expression quantification of *TGF-β*, *Runx2*, and *ALPL* by human osteoblasts Saos-2 treated with non-cytotoxic HT-NE concentrations after 1, 3, and 7 days. The *TGF-β* expression was significantly increased after the treatment with HT-NE 64 µM at 3 and 7 days (*p* < 0.05, *p* < 0.001). *Runx2* gene expression showed a statistically significant increase after all tested periods when treated with HT-NE 32 µM (*p* < 0.05, *p* < 0.001, *p* < 0.0001). At the 64 µM concentration of HT-NE, the increase in its expression was observed at 3 and 7 days (*p* < 0.05). The concentration of HT-NE 128 µM showed an increased effect of *Runx2* gene expression just after 7 days (*p* < 0.01). Finally, the expression of the *ALPL* gene showed a statistically significant increase after the treatment with HT-NE 32 µM at 1 and 7 days (*p* < 0.01, *p* < 0.0001). Additionally, after 7 days HT-NE 64 µM and 128 µM showed a statistically significant increase in the expression of this gene (*p* < 0.05, *p* < 0.0001).

#### 3.2.6. Collagen Production by Picrosirius Red

**Figure 8:**
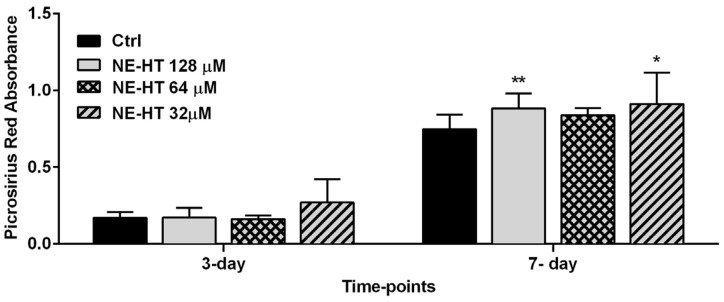
Collagen production quantification after treatment with non-cytotoxic HT-NE concentrations (128 µM, 64 µM, and 32 µM) for 3 and 7 days. Asterisk: Represents a statistically significant difference compared to the control group (one-way ANOVA, * *p* < 0.05, ** *p* < 0.01).

Figure 8 shows the collagen quantification after treatment with different HT-NE concentrations (128 µM, 64 µM, and 32 µM) for 3 and 7 days. The results showed that at 3 days of treatment, HT-NE treated groups did not show a statistical difference compared to the control group (*p* > 0.05). However, at 7 days of treatment, the HT-NE at 128 µM and 32 µM showed an increase in collagen production that was statistically significant compared to the control group (*p* < 0.05).

#### 3.2.7. Alkaline Phosphatase Activity

**Figure 9:**
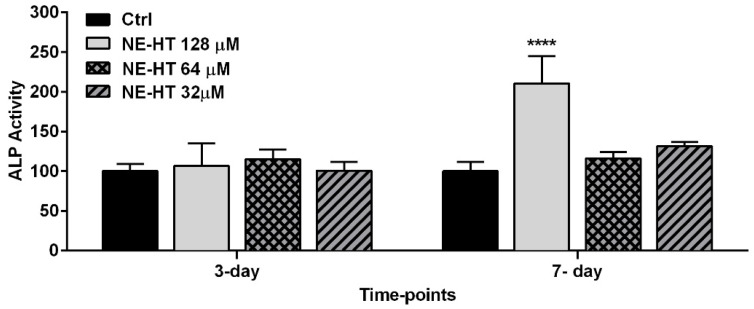
Alkaline phosphatase activity at the periods of 3 and 7 days after the treatment of osteoblasts Saos-2-like with HT-NE 128 µM, 64 µM, and 32 µM. Asterisk: represents a statistically significant difference compared to the control group (ANOVA, **** *p* < 0.0001).

Figure 9 shows the results of the ALP activity analysis. It is possible to note that after the treatment with all HT-NE concentrations, no statistical difference between groups was observed at 3 days of treatment (*p* > 0.0001). On the other hand, we can observe that at 7 days the treatment with 128 µM of HT-NE showed a statistically significant increase in ALP activity compared to the control group (*p* < 0.0001).

## 4. Discussion

Bone diseases are a recurring problem in the general population and are presented in diverse ways leading to mass bone loss [1]. New treatments are studied to solve this problem with less collateral effect and good efficacy [31]. In this way, we hypothesize that a new formulation of HT-NE may permit a controlled release of HT and provide safe treatment techniques, in addition to enhancing its biocompatibility and biological activities. The focus of the present study was to develop a new HT-NE and characterize it through the evaluation of droplet size, polydispersity index, and zeta potential to guarantee its colloidal stability and its correct function. Additionally, we investigated its effects on bone metabolism through different analyses in human osteoblasts Saos-2-like, such as cell proliferation, calcium nodule formation, gene expression, collagen production, and ALP activity.

A droplet size smaller than 100 nm can reduce the interfacial tension between the oil phase and water, preventing the droplets’ coalescence and maintaining their disposition and distribution [32]. Additionally, NE with a small droplet size presents trans-lucence or slight turbidity [33] as observed in our formulations. Regarding the PDI, values less than 0.5 are ideal to obtain a good dispersion, ensuring the formulation’s homogeneity [34]. Taking this into account, the new HT-NE development in the present study showed suitable parameters with mean values of PDI of 0.267 ± 0.008 for 21 days. In a previous study by Bosly [35], in 2022, an NE using a high sonication method to obtain nanoparticles was prepared, similar to our study. The authors confirmed the homogeneity, stability, and adequate particle distribution of the NE with a PDI of 0.342, which was optimized, acquiring a PDI of 0.263 [35]. The outcome of our 90-day characterization period reveals a PDI of 0.584. While these data do not meet the criteria for an ideal index, they demonstrate proximity to the standard for NE, showing an increase in PDI directly correlated with time [36]. Furthermore, the trend of increasing rates over time underscores the stability of the formulation within shorter periods.

The zeta potential is another important characteristic of the development of a new NE because it will generate the necessary repulsive potential to prevent the coalescence of the droplets [37]. Since the components of the formulation such as capric/caprylic acid triglycerides and polysorbate 80 do not possess any ionizable groups at pH 7 [38,39] the negative zeta potential is likely a result of residual negative charges attributed to the phosphate groups the phospholipid soy phosphatidylcholine, which can ionize at pH 7. The drug HT also lacks ionizable groups at pH 7 [40]. However, when adding a non-charged molecule to a predominantly negatively charged system, the zeta potential will be partially neutralized, thereby decreasing, as observed [37]. Due to the low water solubility of HT and the formation of nano-droplets within NE, a small fraction of HT could be situated at the oil/water interface, while the biggest fraction of HT is more likely located within the oily core of NE droplets due to its lipophilicity [41]. In addition to that, the zeta potential will affect directly the NE capacity of permeability in cell membranes, since these are negatively charged, and request a specific cationic and anionic balance to allow biocompatibility, in order to reach this biocompatibility as better as possible, the ideal values of zeta potential usually is kept less than −30 mV and more than 30 mV [42]. Our HT-NE presented values of zeta potential between 20 mV and −20 mV, showing compatibility with studies that obtained these same values and resulted in the stability of the nanoemulsion [43].

To evaluate the amount of the main compound present in the HT-NE, the encapsulation efficiency was performed. Our results showed an encapsulation efficiency of 74 ± 5.33% demonstrating the great capacity of the NE to carry the HT. Similar results of encapsulation efficiency were shown by Duranoğlu et al., 2018 [44], who developed a nanoparticle system using HT and obtained an encapsulation efficiency of 80 ± 4.9%. However, they obtained a minimum particle size of 260.2 ± 16.5 nm, showing that our HT-NE allowed optimization nanoparticle system, maintaining a good encapsulation and droplet size. In addition, it was demonstrated that the use of polysorbate 80 as a surfactant enables an encapsulation efficiency of 80.7 ± 7.15% and satisfactory results of characterization [45].

The release’s speed can be affected by diverse factors, such as the particle size, viscosity, compound solubility, and release matrix interaction with the active compound [46]. A quick dissolution of the compound in the solution can be followed by a facilitated diffusion [46]. The presence of an NE can act as a barrier to the immediate release of the compound, allowing a controlled release over time. The NE structure can supply extra protection to HT, extending this way, its release [47]. The presented results are accordingly to previous studies that highlighted the influence of a formulation and its physiochemical structure on the release kinetics of compounds [46,48]. Understanding these factors is crucial for the development of efficient pharmaceutical formulations and the optimization of the controlled release of compounds, such as HT, aiming for future therapeutic applications. When our results are compared to founds for Diedritch et al., in 2023, where they encapsulated luteolin, a flavonoid from the same group of HT, their NE showed a release of 50% in 12 h [49]. Our study demonstrates a rapid release within 10 h, which may be attributed to its higher viscosity, rendering it a promising therapy for local applications, since it presents a controlled release that does not take a long time, and formulates properly to systemic functions. Taking into account the mathematical models, in the consistent Korsmeyer–Peppas model, used in various studies [50,51], the k and n values, in this context, represent the release rate to the encapsulated compound through the release system and mean important parameters in the drug release from the nanoparticle system [50]. This way, the obtained n value of 0.7485 indicates that the HT release from the NE probably is following a non-Fickian or anomalous diffusion mechanism, suggesting that the release process is influenced not only by the compound diffusion but also by other factors, such as the release matrix structure and the HT and NE interaction.

In terms of possible clinical use, the cytotoxicity test is extremely important for the evaluation of new therapeutic drug delivery systems. Thus, the cytotoxicity of our new HT-NE was evaluated on osteoblasts Saos-2, which are important cells present in bone metabolism. The cells exposed for 7 days to the HT-NE and Free HT, showed a concentration-dependent effect. The HT-NE, like all nanoemulsions, presents a complex composition responsible for bringing more benefits as a differential delivery of drugs and a controlled release, avoiding compound oxidation and other destructive processes [52,53]. For this reason, is expected that its biocompatibility is in lower concentrations than the free compound. In addition to that, high concentrations could be used for osteogenic effects analysis, demonstrating promising results.

Our formulation did not present cytotoxic effects in concentrations less than 256 µM, showing interesting enhancement of the biocompatibility when compared with Xue et al. [21] in 2017, which showed an inhibition of pre-osteoblastic cell proliferation with HT treatment at 100 µM. Also, Blank NE presents a non-cytotoxic effect in concentrations less than 512 µM. In contrast, the Blank NE developed by Vaz et al. [54], was formulated similarly to ours, using a high-energy method, but using castor oil instead of capric/caprylic acid. The authors showed a reduction of nasal cavity cell viability (around 80%) with the Blank NE at 131 µM [21]. Since there are not many studies showing osteoblasts and NE biocompatibility, we can suggest that the new formulation and its chemicals combination developed in the present study showed excellent parameters of biocompatibility.

Osteoblast cells have a very important role in bone formation and differentiation [55]. The evaluation of cell proliferation is an important marker to show if our formulation has a good effect on the cells that are being treated and confirm its biocompatibility [55]. An enhancement in osteoblast cell metabolism can offer the possibility of the positive effect of HT-NE on bone metabolism. There are few studies regarding encapsulating in a nanosystem and evaluation of the biological properties of the HT using osteoblasts test models, and none of them evaluated cell proliferation. On the other hand, Trzeciakiewicz et al., in 2009 [20] showed in their study that the Free HT did not affect the primary rat osteoblasts proliferation in the highest non-cytotoxic concentration (10 µM). This study corroborates the hypothesis that the encapsulation of HT in an NE can enhance its properties.

In osteogenesis, many processes are involved from the beginning of formation until the maturation of bone. Between them, an important step is the calcium nodule mineralization by osteoblasts, which generates the production of hydroxyapatite, the inorganic compound responsible for bone formation [56]. Our formulation showed an increase in the mineralization in all concentrations evaluated, which contrasts with a previous study that tested the HT in its free form and did not find this effect [20]. A previous study evaluated the influence of HT and an HT nanocrystal in calcium nodule formation after 3 weeks of treatment [57]. The authors showed an increase in nodule formation after the treatment with HT and a better effect by HT nanocrystals [57]. These previous results are in accordance with ours, because we demonstrated that HT-NE was also able to promote this increase, but in a shorter period (7 days), giving a hypothesis of the acceleration of this function.

On the other hand, the ALP activity had a correlation with nodule mineralization [58], because the increase in alkaline phosphatase is essential to the beginning of nodule formation. In the present study, at the period of 7 days, the HT-NE presents a great effect in the increase in ALP activity and shows the nodule formation increase. Trzeciakiewicz et al. [23] showed that the ALP activity of Free HT was highly expressed at 14 days of treatment in primary rat osteoblasts. Our results are in accordance with these authors because Free HT and HT encapsulated in an NE can express high levels of ALP in early periods of mineralization.

Bone-specific gene expression patterns are highly involved in the bone formation process, preceding the osteoblasts differentiation, or during the maturation and proliferation of these cells [59,60,61,62]. *Runx2* is one of the first transcription factors expressed in mesenchymal cells, leading to osteoblast formation and maturation [60,61]. The *ALPL* (alkaline phosphatase) gene is responsible for bone maturation keeping its density and avoiding dysplasia [62] and *TGF-β*, is a gene responsible for cell survivor, proliferation and differentiation, leading to bone growth through the BMP9 signaling pathway [63]. Their roles in bone metabolism elucidate the understanding of our formulation action. Similar to our results, Trzeciakiewicz et al. [20], showed a time-dependent effect of HT in the expression of genes *Runx2* and *ALPL* using primary rat osteoblasts. On the other hand, it was shown that the deficiency of *TGF-β* in mouses led to a reduction in bone growth and mineralization [64]. Regarding the effect of HT or a new NE associated with HT on *TGF-β* expression, we demonstrate for the first time the positive effect of this flavonoid on this gene expression.

Finally, collagen is an essential protein in bone composition. The collagen fiber associated with the hydroxyapatite is responsible for bone formation, being capable of elevating bone mass [65]. For this reason, many biocompatible materials are being developed aiming to enhance and increase collagen production [66]. As shown in the results section, at 7 days our formulation showed an increase in collagen production, corroborating the hypothesis that the HT-NE has a promising effect on osteogenesis, mainly in the beginning of osteoblasts maturation, allowing the enhancement of bone density. In addition to that, a study using the HT in its free form did not find an influence on collagen production [23]. Taking these results into account, the encapsulation of HT in our NE improved its biological effect, specifically on collagen production.

Despite the limitations of the present study, new in vitro and in vivo models can provide other answers and complement our results to future medical applications. More numbers of research works are relevant to optimize the properties of the HT-NE, as well as evaluate the clinical performance of this new biomaterial. Such as the biocompatibility with other cell types, for example, mesenchymal stem cells, since they are progenitor cells for different types of cells, fibroblasts, macrophages, and keratinocytes, and the efficacy and collateral effects of our formulation in vivo. In addition to that, we note that the HT-NE developed in the present study did not present a dose-dependent effect in our results. Then, there are still numerous questions about HT-NE and new studies are necessary to corroborate our findings.

## 5. Conclusions

The results obtained indicate satisfactory parameters of characterization and promisor biological performance of the novel HT-NE. The new formulation allowed the incorporation of HT with similar non-cytotoxic concentrations with Free HT, in addition to its lower IC_50_ values. In addition to that, the present results show that the HT-NE can be applied in high concentrations in the cell culture, enhancing the HT properties, allowing an increase or acceleration of its effects on bone metabolism, through the increase in cell proliferation, calcium nodule formation, regulation of important genes to bone metabolism, ameliorate the collagen production and ALP activity. In this way, we consider this new biomaterial to be a promising treatment for bone diseases. Although more in vitro and in vivo studies are necessary.

## Figures and Tables

**Table 1:** Droplet size, polydispersity index, and zeta potential of HT-NE and Blank NE.

	Time (Days)	Droplet Size (nm)	Polydispersity Index	Zeta Potential (mV)
	1	88.79 ± 0.09	0.276 ± 0.003	−13.27 ± 2.7
	7	88.26 ± 0.75	0.273 ± 0.006	−17.37 ± 1.7
HT-NE	21	89.01 ± 1.15	0.266 ± 0.003	−14.97 ± 0.6
	90	82.11 ± 3.89	0.584 ± 0.035	−4.75 ± 0.1
Blank NE	1	97.86 ± 0.50	0.278 ± 0.003	−26.9 ± 2.4

**Table 2:** Mathematical release kinetics models for HT-NE.

	NE		
Model	k	R^2^	n
Zeroth order	0.0205	0.8827	
First order	0.2142	0.9545	
Higuchi	28.88	0.9438	
Hixson–Crowel	0.0585	0.9699	
Korsmeyer–Peppas	18.21	0.9912	0.7485

## Data Availability

Data are contained within the article.
